# Role of ANGPTL8 in NAFLD Improvement after Bariatric Surgery in Experimental and Human Obesity

**DOI:** 10.3390/ijms222312945

**Published:** 2021-11-30

**Authors:** Carolina M. Perdomo, Javier Gómez-Ambrosi, Sara Becerril, Víctor Valentí, Rafael Moncada, Eva M. Fernández-Sáez, Leire Méndez-Giménez, Silvia Ezquerro, Victoria Catalán, Camilo Silva, Javier Escalada, Gema Frühbeck, Amaia Rodríguez

**Affiliations:** 1 Department of Endocrinology & Nutrition, Clínica Universidad de Navarra, 31008 Pamplona, Spain; cperdomo@unav.es (C.M.P.); csilvafr@unav.es (C.S.); fescalada@unav.es (J.E.); gfruhbeck@unav.es (G.F.); 2 Metabolic Research Laboratory, Clínica Universidad de Navarra, 31008 Pamplona, Spain; jagomez@unav.es (J.G.-A.); sbecman@unav.es (S.B.); efernandezs.1@alumni.unav.es (E.M.F.-S.); lmgimenezde@gmail.com (L.M.-G.); silvia_ezquerro@hotmail.com (S.E.); vcatalan@unav.es (V.C.); 3 CIBER Fisiopatología de la Obesidad y Nutrición (CIBEROBN), Instituto de Salud Carlos III, 28029 Madrid, Spain; vvalenti@unav.es (V.V.); rmoncada@unav.es (R.M.); 4 Obesity and Adipobiology Group, Instituto de Investigación Sanitaria de Navarra (IdiSNA), 31008 Pamplona, Spain; 5 Department of Surgery, Clínica Universidad de Navarra, 31008 Pamplona, Spain; 6 Department of Anesthesia, Clínica Universidad de Navarra, 31008 Pamplona, Spain

**Keywords:** NAFLD, obesity, bariatric surgery, lipogenesis, angiopoietin-like protein 8

## Abstract

Angiopoietin-like protein 8 (ANGPTL8) is an hepatokine altered in several metabolic conditions, such as obesity, type 2 diabetes, dyslipidemia and nonalcoholic fatty liver disease (NAFLD). We sought to explore whether ANGPTL8 is involved in NAFLD amelioration after bariatric surgery in experimental models and patients with severe obesity. Plasma ANGPTL8 was measured in 170 individuals before and 6 months after bariatric surgery. Hepatic ANGPTL8 expression was evaluated in liver biopsies of patients with severe obesity undergoing bariatric surgery with available liver pathology analysis (*n* = 75), as well as in male Wistar rats with diet-induced obesity subjected to sham operation, sleeve gastrectomy or Roux-en-Y gastric bypass (RYGB) (*n* = 65). The effect of ANGPTL8 on lipogenesis was assessed in human HepG2 hepatocytes under palmitate-induced lipotoxic conditions. Plasma concentrations and hepatic expression of ANGPTL8 were increased in patients with obesity-associated NAFLD in relation to the degree of hepatic steatosis. Sleeve gastrectomy and RYGB improved hepatosteatosis and reduced the hepatic ANGPTL8 expression in the preclinical model of NAFLD. Interestingly, ANGPTL8 inhibited steatosis and expression of lipogenic factors (*PPARG2*, *SREBF1*, *MOGAT2* and *DGAT1*) in palmitate-treated human hepatocytes. Together, ANGPTL8 is involved in the resolution of NAFLD after bariatric surgery partially by the inhibition of lipogenesis in steatotic hepatocytes.

## 1. Introduction

Nonalcoholic fatty liver disease (NAFLD) is the most common cause of chronic liver disease and comprises a spectrum of liver disorders ranging from simple steatosis to nonalcoholic steatohepatitis (NASH), fibrosis and cirrhosis [1]. The incidence of NAFLD is rising due to the pandemic spread of obesity and type 2 diabetes (T2D). In this regard, the global prevalence of NAFLD in the general population is 24% [2], increasing to 75–90% and 55% in patients with severe obesity and T2D, respectively [3,4]. Bariatric surgery induces a long-term maintenance of weight loss, along with the improvement of insulin resistance and T2D remission, in patients with severe obesity [5,6]. Moreover, bariatric surgery has been proposed as a therapeutic option to reduce liver injury in those patients with obesity who have been diagnosed for NAFLD and are unresponsive to lifestyle interventions and pharmacotherapy [7,8]. Sleeve gastrectomy and Roux-en-Y gastric bypass (RYGB), the most commonly used bariatric surgical procedures, appear to be equally efficacious in the improvement of liver transaminases and histological lesions in patients with obesity and NAFLD [9]. Nonetheless, solid and mechanistic data on the comparative effects of these techniques on NAFLD amelioration are still needed.

Angiopoietin-like protein 8 (ANGPTL8), also known as lipasin, RIFL, C19orf80, TD26 or betatrophin, is a nutritionally-regulated hepatokine that is downregulated during fasting and upregulated after feeding [10]. Loss- and gain-of-function studies on mice revealed that ANGPTL8 constitutes a critical regulator of lipid metabolism. Adenoviral overexpression of the *ANGPTL8* gene in mice results in an increase in plasma triacylglycerol (TG) levels [10] and, consistently, ANGPTL8-knockout mice exhibit low TG concentrations [11,12]. In humans, ANGPTL8 interacts with ANGPTL3 or ANGPTL4 to inhibit LPL activity [10,13] and several *ANGPTL8* gene variants have been linked to lipid alterations [14,15,16]. Interestingly, serum ANGPTL8 concentrations are decreased in several metabolic conditions, such as obesity, T2D or dyslipidemia [17,18,19] and increase after bariatric surgery [20], although contradictory results have been published [21]. By contrast, increased circulating and hepatic expression of ANGPTL8 levels are positively associated with different stages of NAFLD [22,23,24,25]. In this context, the aim of the present study was to evaluate whether ANGPTL8 is associated with the improvement of hepatic steatosis after sleeve gastrectomy and RYGB in a cohort of patients with severe obesity and different degrees of insulin resistance, as well as in experimental models of diet-induced obesity. Moreover, the direct effects of ANGPTL8 on key regulatory molecules involved in lipogenesis were also studied in vitro in human HepG2 hepatocytes under palmitic acid-induced lipotoxic conditions.

## 2. Results

### 2.1. Prevalence of NAFLD and Metabolic Improvement after Bariatric Surgery in the Studied Population

Anthropometric and clinical variables of the patients with severe obesity included in the study are shown in Table 1. As expected, all indices of adiposity (body mass index (BMI), waist circumference, body fat percentage and serum leptin levels) were higher (*p* < 0.0001) in participants with severe obesity compared with normal-weight controls. Increased (*p* < 0.05) markers of insulin resistance, evidenced by glycemia and insulinemia at baseline levels and 2 h after an oral glucose tolerance test (OGTT), homeostasis model assessment (HOMA), quantitative insulin sensitivity check index (QUICKI) and adipocyte insulin resistance index (Adipo-IR), were detected in patients with impaired glucose tolerance (IGT) and T2D compared with those with normoglycemia (NG). A more detrimental lipid profile, systemic inflammation and hepatic function were also detected in patients with insulin resistance. The prevalence of biopsy-proven NAFLD in our patients with severe obesity was 80.6%, being higher (*p* < 0.0001) in men (95.0%) than in women (73.8%). Biopsy-proven NASH was detected in 41.9% of the total sample, and fibrosis was found in 23.2% of liver specimens. Individuals with insulin resistance exhibited higher (*p* < 0.05) prevalence rates of NAFLD (NG 66.0%, IGT 86.4% and T2D 94.5%) and NASH (NG 34.0%, IGT 45.9% and T2D 48.0%). No patient had evidence of established cirrhosis. After an average of 6 months after bariatric surgery, patients with severe obesity undergoing sleeve gastrectomy or RYGB experienced a profound decrease in body weight, whole-body adiposity and leptinemia and an improvement in insulin sensitivity, as well as a decrease in γ-glutamyltransferase (γ-GT) levels (Table 2). A better lipid profile and an improved systemic inflammation was observed in patients with severe obesity submitted to RYGB compared to those undergoing sleeve gastrectomy.

### 2.2. Plasma Concentrations of ANGPTL8 Are Increased in Obesity-Associated NAFLD

**Figure 1:**
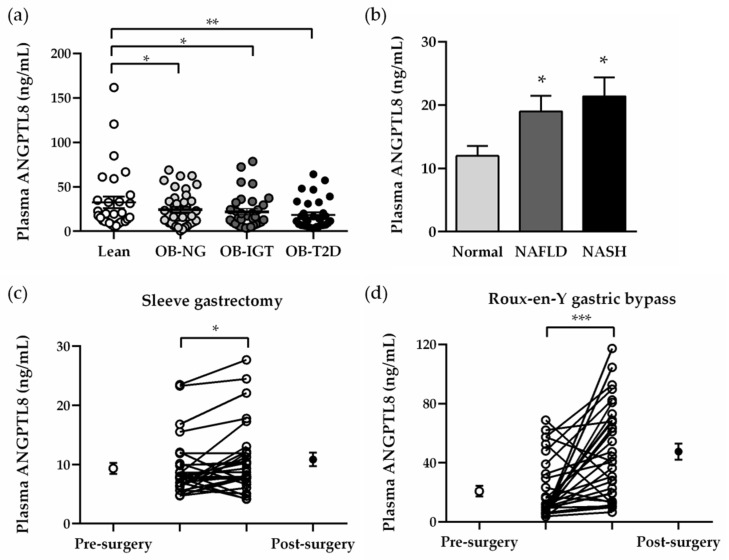
Effect of insulin resistance and NAFLD on plasma concentrations of ANGPTL8 in patients with severe obesity before and after bariatric surgery. Fasting plasma concentrations of ANGPTL8 according to severe obesity and insulin resistance (**a**) or hepatic function (**b**). Impact of weight loss achieved 6 months after sleeve gastrectomy (**c**) or RYGB (**d**) in plasma ANGPTL8 levels. Statistical differences were analyzed by one-way ANOVA followed by a Tukey’s test or by a two-tailed paired Student’s *t*-test, where appropriate. * *p* < 0.05; ** *p* < 0.01; *** *p* < 0.001 vs. patients with NG or normal liver.

Obesity was associated with low plasma ANGPTL8 concentrations (normal weight 32.45 ± 5.57 vs. obesity 19.69 ± 1.49 ng/mL, *p* = 0.004), regardless of the degree of insulin resistance (Figure 1a). Biopsy-proven NAFLD in patients with obesity was related to increased circulating levels of ANGPTL8 (normal liver 11.94 ± 1.60 vs. NAFLD 20.00 ± 1.96 ng/mL, *p* = 0.003) in both stages of NAFLD and NASH (Figure 1b). Plasma ANGPTL8 levels were positively correlated with HDL cholesterol concentrations (r = 0.24, *p* = 0.004), while negatively correlated with hepatic function marker γ-GT (r = −0.24, *p* = 0.019) after age and sex adjustments. Multivariate analyses were performed in order to detect the association between plasma ANGPTL8 with variables related to insulin resistance (*Model I*) and liver function and fat percentage (*Model II*) (Table S2). Both HOMA and γ-GT contributed independently to circulating ANGPTL8 after controlling for age, sex and BMI. Six months after bariatric surgery, an increase in plasma ANGPTL8 was observed in patients with obesity submitted to sleeve gastrectomy (pre-surgery 9.34 ± 0.94 vs. post-surgery 10.85 ± 1.13 ng/mL, *p* < 0.05) and RYGB (pre-surgery 20.76 ± 3.53 vs. post-surgery 47.53 ± 5.47 ng/mL, *p* < 0.001).

### 2.3. Hepatic Expression of ANGTL8 Is Upregulated in Patients with Morbid Obesity and NAFLD in Relation to Their Degree of Liver Steatosis

**Figure 2:**
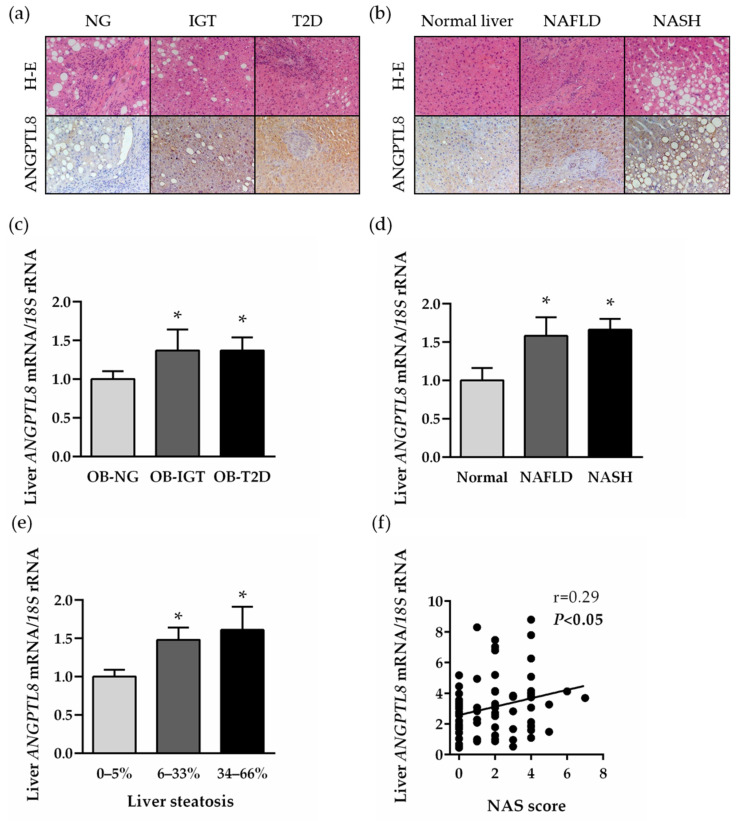
Hepatic ANGPTL8 expression in obesity-associated T2D and NAFLD. Liver sections obtained from patients with severe obesity classified according to their degree of insulin resistance (**a**) or hepatic function (**b**) stained with H-E (*upper panels*) and marked with antibodies against ANGPTL8 (*lower panels*) (magnification 200×). Bar graphs show the effect of insulin resistance (**c**), NAFLD (**d**) and the degree of liver steatosis (**e**) on hepatic *ANGPTL8* transcripts. (**f**) Correlation between hepatic *ANGPTL8* mRNA and NAS score. Statistical differences were analyzed by one-way ANOVA followed by a Tukey’s test. * *p* < 0.05 vs. patients with NG, normal liver or 0–5% liver steatosis.

The presence of ANGPTL8 in the human liver was evaluated in liver biopsies (*n* = 74) obtained from patients with morbid obesity by immunohistochemistry and real-time PCR. The tissue distribution of ANGPTL8 was mainly detected in hepatocytes, and the immunostaining was markedly increased in liver sections obtained from patients with insulin resistance (Figure 2a) and NAFLD (Figure 2b). Accordingly, the transcription of the *ANGPTL8* gene was upregulated in parallel with the impairment of insulin sensitivity (Figure 2c) and hepatic function (Figure 2d). Consistent with these findings, *ANGPTL8* mRNA levels were increased according to the degree of steatosis (Figure 2e) and were positively correlated with the NAFLD activity score (NAS) (Figure 2f). Moreover, multiple regression analysis revealed that the liver steatosis contributed independently to 41.8% of variation in hepatic *ANGPTL8* transcripts after controlling for age, sex and BMI.

### 2.4. Increased Hepatic Expression of ANGPTL8 in a Preclinical Model of Diet-Induced Obesity Is Downregulated after Bariatric Surgery

**Figure 3:**
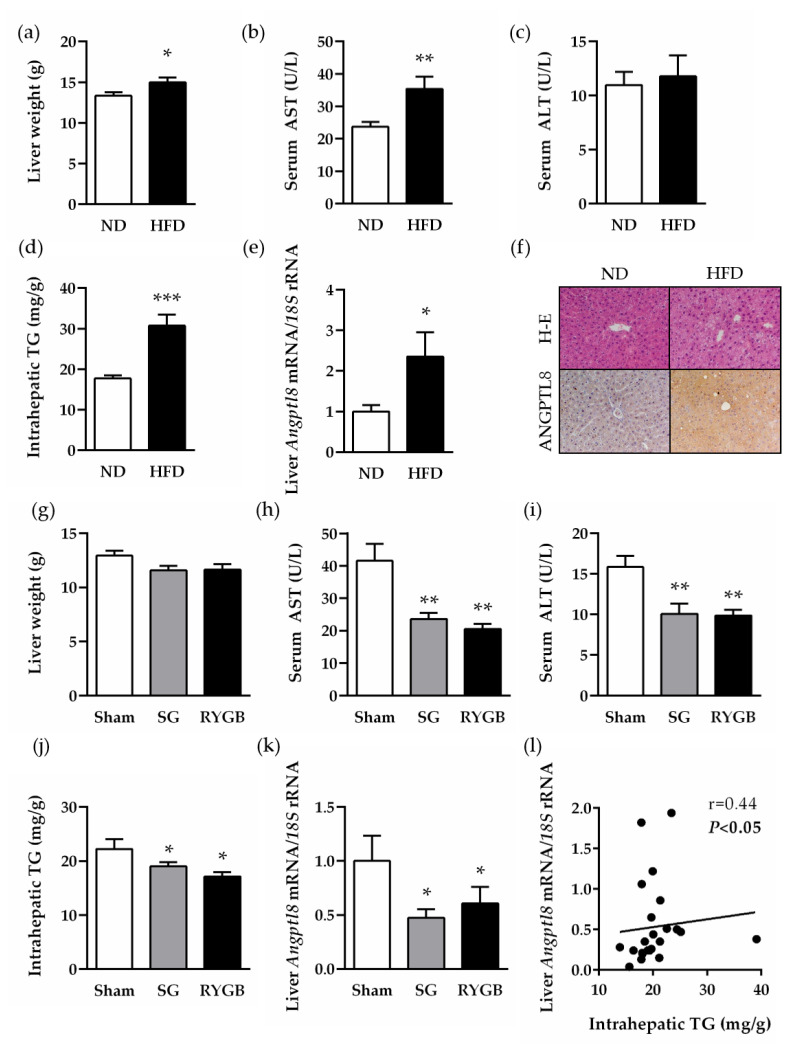
Hepatic expression of ANGPTL8 in rats with diet-induced obesity before and after bariatric surgery. Bar graphs show the effect of diet-induced obesity and weight loss achieved 1 month after sleeve gastrectomy (SG) or Roux-en-Y gastric bypass (RYGB) on rat liver weight (**a**,**g**), AST (**b**,**h**) and ALT (**c**,**i**) transaminase levels, intrahepatic TG content (**d**,**j**) and hepatic *Angptl8* transcript levels (**e**,**k**). (**f**) Liver sections stained with H-E (*upper panels*) and marked with antibodies against ANGPTL8 (*lower panels*) (magnification 200x). (**l**) Correlation between post-surgical hepatic mRNA expression of *ANGGPTL8* and intrahepatic TG. Statistical differences were analyzed by a Student’s *t*-test or one-way ANOVA followed by a Tukey’s test, where appropriate. * *p* < 0.05, ** *p* < 0.01 and *** *p* < 0.001 vs. rats fed a ND or sham-operated groups.

To corroborate the human findings regarding the potential involvement of ANGPTL8 in the improvement of NAFLD after bariatric surgery, an experimental model of diet-induced obesity submitted either to sleeve gastrectomy or RYGB was used. As expected, animals on a high-fat diet (HFD) for 4 months exhibited an increased body weight and adiposity, as well as impaired glucose tolerance and insulin resistance, compared to those fed a normal diet (ND) (Figure S1). Diet-induced obesity was also associated with impaired liver function and steatosis, evidenced by higher liver weight and serum AST levels, as well as an increased intrahepatic triacylglycerol content (all *p* < 0.05) (Figure 3a–d). Transcript levels of rat *ANGPTL8* were significantly increased (*p* < 0.05) in the liver of rats with diet-induced obesity compared to lean control rats (Figure 3e). Accordingly, a strong immunostaining for the ANGPTL8 protein was observed in hepatocytes of liver sections obtained from rats fed a HFD (Figure 3f). One month after bariatric surgery, obese animals submitted either to sleeve gastrectomy or RYGB showed an improved body weight, adiposity and metabolic profile (Figure S2). Both bariatric surgical procedures significantly reduced serum AST and ALT transaminase levels and liver steatosis (all *p* < 0.05) in obese rats, without changes in liver weight (Figure 3g–j). Importantly, hepatic *ANGPTL8* mRNA was significantly downregulated after sleeve gastrectomy and RYGB, and a positive association of post-surgical *ANGPTL8* transcript levels with liver steatosis (Figure 3l) and AST (r = 0.57, *p* = 0.002) was detected.

### 2.5. ANGPTL8 Inhibits Palmitate-Induced Lipogenesis, but Not Its Anti-Proliferative Action, in Human HepG2 Hepatocytes

**Figure 4:**
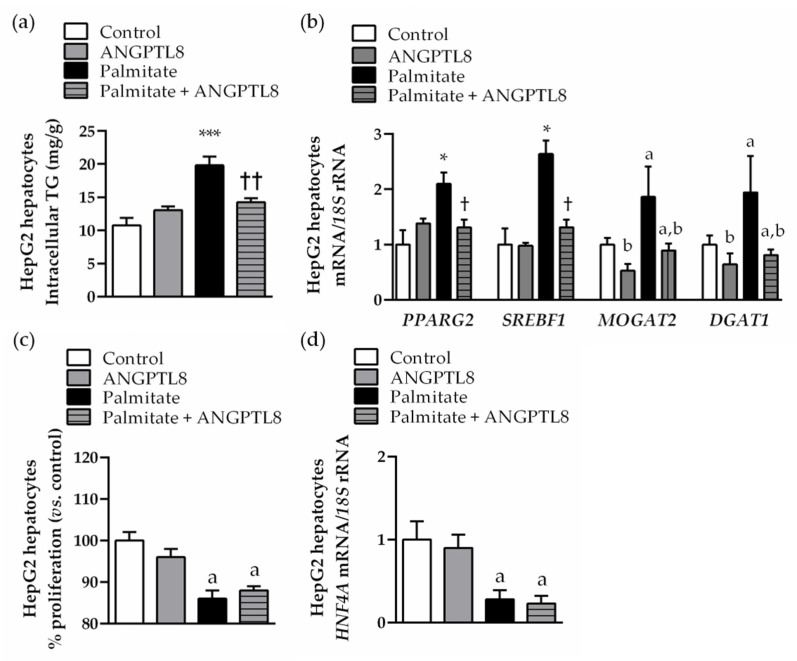
ANGPTL8 reversed palmitate-induced lipogenesis in human HepG2 hepatocytes. Bar graphs show the (**a**) intracellular triacylglycerol content and (**b**) mRNA expression of lipogenic transcription factors and enzymes in HepG2 hepatocytes stimulated with palmitate in the absence or presence of ANGPTL8 for 24 h. Proliferation rate (**c**) and transcript levels of hepatocyte differentiation transcription factor *HNF4A* (**d**) are shown. Statistical differences were analyzed by a two-way ANOVA or a one-way ANOVA followed by Tukey’s post hoc test, if an interaction between factors was detected. * *p* < 0.05; *** *p* < 0.001 vs. unstimulated HepG2 cells; † *p* < 0.05; †† *p* < 0.01 vs. palmitate-treated HepG2 cells; a *p* < 0.05 effect of palmitate; b *p* < 0.05 effect of ANGPTL8.

The direct effect of ANGPTL8 against lipotoxicity was tested in an in vitro model of NAFLD. Human HepG2 hepatocytes were exposed to palmitate (200 μmol/L) in the absence or presence of ANGPTL8 (10 ng/mL) for 24 h. As expected, palmitate stimulation increased (*p* < 0.05) intracellular TG content (Figure 4a) and upregulated (*p* < 0.05) lipogenic transcription factors (*PPARG2* and *SREBF1*) and enzymes (*MOGAT2* and *DGAT1*) (Figure 4b) in HepG2 hepatocytes. ANGPTL8 reduced the steatosis (*p* < 0.05) and mRNA expression levels of *PPARG2, SREBF1, MOGAT2* and *DGAT1* lipogenic genes of palmitate-treated hepatocytes (Figure 4a,b). However, ANGPTL8 could not prevent the anti-proliferative action of palmitate in steatotic HepG2 hepatocytes, evidenced by a lower proliferation rate (Figure 4c) and decreased mRNA expression of the master regulator of hepatocyte differentiation *HNF4A* compared to control cells (Figure 4d).

## 3. Discussion

ANGPTL8 is an hepatokine regulated by nutritional status that plays an important role in lipid metabolism [21]. In the fed state, hepatic expression of ANGPTL8 is increased and ANGPTL8 forms hetero-complexes with ANGPTL3 in the liver and with ANGPTL4 in the adipose tissue to inhibit LPL, an enzyme that hydrolyzes TG from chylomicrons and VLDL into FFA [26]. By contrast, during fasting, circulating ANGPTL8/ANGPTL3 complexes are reduced, thereby increasing LPL activity in peripheral tissues to ensure the energy supply [27]. ANGPTL8 is upregulated by pro-lipogenic stimuli, such as insulin or glucose, in the adipose tissue [28] but, to our knowledge, the direct action of this hepatokine on lipogenesis has not yet been explored in the liver. HepG2 hepatocytes are widely used to model human NAFLD in vitro when exposed to oleic or palmitic acid leading to steatosis and apoptosis [29]. We herein show that, in palmitate-induced human HepG2 hepatocytes, ANGPTL8 decreased intracellular TG content by downregulating the transcript levels of the lipogenic transcript factors (*PPARG* and *SREBF1*) and enzymes (*MOGAT2* and *DGAT1*). The relationship between ANGPTL8 and NAFLD remains unclear, with controversial reports indicating increased [23,30,31] or decreased [32] circulating levels of ANGPTL8 in patients with biopsy-proven NAFLD compared with healthy volunteers. In our hands, plasma ANGPTL8 concentrations were elevated in obesity-associated NAFLD and negatively correlated with HDL cholesterol, confirming the inseparable association between ANGPTL8 and lipid disorders [19,33]. Furthermore, hepatic mRNA and protein expression of ANGPTL8 was upregulated in patients and rat models with obesity-associated NAFLD in relation to the degree of hepatic steatosis. A similar upregulation of hepatic ANGPTL8 was reported in genetically obese *ob*/*ob* and *db*/*db* mice, as well as in mice fed a high-fat or methionine-choline-deficient diet [23]. Together, the increase in ANGPTL8 seems to protect the liver against lipotoxicity partially through the direct inhibition of lipogenesis in human steatotic hepatocytes.

Bariatric surgery improves serum transaminases, NAS score and NAFLD fibrosis score in patients with severe obesity, with sleeve gastrectomy and RYGB being equally effective in ameliorating NAFLD [9,34,35]. In line with this observation, our data confirmed that sleeve gastrectomy and RYGB ameliorates hepatic function, as evidenced by an improved profile of AST and ALT and hepatosteatosis in an experimental model of NAFLD. Interestingly, sleeve gastrectomy and RYGB induced a downregulation of ANGPTL8 in the liver of diet-induced obese rats 1 month after surgical interventions. Moreover, post-surgical hepatic *ANGPTL8* transcripts were inversely related to liver TG content and transaminase AST, supporting the hepatoprotective action of ANGPTL8 against liver steatosis. In line with this observation, metabolic adaptations after bariatric surgery include modulations in the production of hepatokines, such as fetuin-A, selenoprotein P, adropin, sex-hormone-binding globulin (SHBG), insulin-like growth factor binding protein (IGFBP)-2 or fibroblast growth factor 21 (FGF-21), but literature on patients is scarce [36,37,38]. The results of the impact of bariatric surgery on hepatokine ANGPTL8 levels are not univocal. Serum ANGPTL8 levels are increased after sleeve gastrectomy [20,39], although some authors have proposed unique biphasic changes with a first prominent elevation 1 month after sleeve gastrectomy followed by a gradual decrease to reach almost baseline levels 1 year after surgery [39]. Analogously, conflicting results have been published regarding serum ANGPTL8 1 year after RYGB, with some authors observing decreased [40,41], while others increased [20,42], post-surgical levels of this hepatokine. In the present study, we evaluated plasma ANGPTL8 concentrations 6 months after sleeve gastrectomy and RYGB in patients with obesity and biopsy-proven NAFLD. After this post-surgical period, an improved hepatic function evidenced by reduced γ-GT levels was observed, with similar efficacy in both bariatric procedures. Moreover, we found an elevation of ANGPTL8 levels after sleeve gastrectomy and RYGB, suggesting that increased ANGPTL8 levels might be involved in the still ongoing resolution of NAFLD in the medium term after bariatric surgery.

One limitation of the present study constitutes the use of animal models that can lead to translational discrepancies due to species-specific differences when compared to human data. Otherwise, experimental animals are invaluable resources for obesity research, especially as regards the effects of diet on metabolism and disease. In this sense, the use of rat models of diet-induced obesity leading to NAFLD avoids the high inter-individual variability found in patients due to differences in genetics, psychosocial background and pharmacological treatment.

## 4. Conclusions

In conclusion, we herein show that ANGPTL8 inhibits lipogenesis in human hepatocytes exposed to lipotoxic conditions. Moreover, circulating and hepatic ANGPTL8 expression in human and experimental obesity-associated NAFLD were increased in relation to the degree of liver steatosis, and both sleeve gastrectomy and RYGB downregulated the *ANGPTL8* transcripts in the liver of preclinical models. These results support the notion that ANGPTL8 mediates, at least in part, the improvement of NAFLD after bariatric surgery via the improvement of hepatic lipid metabolism.

## 5. Materials and Methods

### 5.1. Patients

Plasma ANGPTL8 was evaluated in a cross-sectional study with 170 samples obtained from normal-weight volunteers (*n* = 30) or patients with severe obesity (*n* = 140) undergoing sleeve gastrectomy or RYGB at the Clínica Universidad de Navarra. Obesity was defined as a BMI ≥ 30 kg/m^2^ and normal weight as a BMI < 25 kg/m^2^. Body composition was measured by air-displacement plethysmography (Bod-Pod^®^, COSMED, Rome, Italy). Patients with obesity were sub-classified into three groups (NG, IGT and T2D) following the criteria of the Expert Committee on the Diagnosis and Classification of Diabetes [43]. Inclusion criteria encompass a complete diagnostic work-up including physical examination, laboratory investigation, ultrasound echography and a liver biopsy consistent with the diagnosis of NAFLD. Exclusion criteria were: (i) significant alcohol abuse (average daily alcohol consumption ≥20 g for women and ≥30 g for men); (ii) the presence of a hepatitis B virus surface antigen or hepatitis C virus antibodies in the absence of a history of vaccination; (iii) use of medication associated with NAFLD within the past year, including amiodarone, valproate, tamoxifen, methotrexate, corticosteroids or anti-retrovirals; (iv) evidence of other specific liver diseases, such as autoimmune liver disease, haemochromatosis, Wilson’s disease or α-1-antitrypsin deficiency evaluated by antinuclear antibody, smooth muscle antibody, ferritin, α-1-antitrypsin and ceruloplasmin levels. In the interventional study, a group of 75 patients with obesity was selected to investigate the impact of weight loss achieved 6 months after sleeve gastrectomy (*n* = 34) or RYGB (*n* = 41) on circulating ANGPTL8. Intraoperative liver biopsies were obtained from patients with severe obesity during sleeve gastrectomy or RYGB to establish a histological diagnosis of the hepatic state, but this procedure is not clinically justified in normal-weight participants. The diagnosis of NAFLD and NASH was established based on Kleiner’s criteria by an expert pathologist masked to all the results of the assays [44]. Features of steatosis, lobular inflammation and hepatocyte ballooning were combined to obtain NAFLD activity score (NAS) (0–8) [44]. All reported investigations were carried out in accordance with the principles of the Declaration of Helsinki, as revised in 2013, approved by the Hospital’s Ethical Committee (protocol 2017.104) and the informed consent from all volunteers was obtained.

### 5.2. Experimental Animals

Four-week-old male Wistar rats (*n* = 65) were fed *ad libitum* during 4 months with either a ND (*n* = 15) (12.1 kJ: 4% fat, 82% carbohydrate and 14% protein, diet 2014 S, Harlan, Teklad Global Diets, Harlan Laboratories Inc., Barcelona, Spain) or a HFD (*n* = 50) (23.0 kJ/g: 59% fat, 27% carbohydrate and 14% protein, diet F3282; Bio-Serv, Frenchtown, NJ, USA). Weight-matched rats with diet-induced obesity were randomly distributed into 3 groups: (a) sleeve gastrectomy (*n* = 10); (b) RYGB (*n* = 6); and (c) sham surgery without gastric resection and intestinal bypass (*n* = 10). Anesthesia, sleeve gastrectomy and RYGB were performed according to previously described methodology [45]. Following the surgical interventions, analgesic care included a subcutaneous administration of 5 mL of buprenorphine (0.03 mg/kg) (Schering-Plough S.A., Madrid, Spain) twice daily for 3 days, as well as topical aluminum powder antiseptic spray over the surgical wound (Aluspray^®^, Vetoquinol UK Ltd., Buckingham, UK). Rats were kept on a liquid diet with 5% glucose and 0.9% saline solution for 3 days and, thereafter, animals were fed a ND. Two weeks after surgery, oral glucose tolerance (OGTT) and intraperitoneal insulin tolerance (IPITT) tests were performed after a 12 h fasting period. Glucose concentrations were measured before and 15, 30, 60, 90 and 120 min after the oral glucose challenge (2 g/kg of body weight) or intraperitoneal insulin administration (0.15 IU/mL) with an automatic glucose sensor (Ascensia Elite, Bayer, Barcelona, Spain). Four weeks after surgical interventions, rats were killed by decapitation after an 8 h fast. All experimental procedures were approved by the Ethical Committee for Animal Experimentation of the University of Navarra (049/10) and conformed to the European Guidelines for the Care and Use of Laboratory Animals (directive 2010/63/EU).

### 5.3. Blood Analysis

Biochemical assays in patients [46] and experimental animals [45] were performed as previously described. The HOMA score of insulin resistance was calculated with the formula: fasting insulin (µU/mL) × fasting glucose (mmol/L)/22.5. An indirect measure of insulin sensitivity was calculated by using the QUICKI index (1/[log(fasting insulin in µU/mL) + log(fasting glucose in mg/dL)]. Adipo-IR index, as a surrogate of adipocyte dysfunction, was calculated as fasting FFA (mmol/L) x fasting insulin (pmol/L). Intrahepatic TG content was measured by enzymatic methods, as previously described [46]. Human plasma ANGPTL8 was determined by ELISA (CSB-EL028107HU, Cusabio, Wuhan, China), with intra- and inter-assay coefficients of variation being <8% and <10%, respectively.

### 5.4. Real-Time PCR

RNA isolation and purification from human and rat liver (25 mg) were performed using TRIzol^®^ Reagent (Invitrogen, Carlsbad, CA, USA) and RNeasy Mini Kit (Qiagen, Maryland, MD, USA), according to manufacturer’s instructions. Transcript levels of human and rat genes encoding *ANGPTL8*, as well as human genes encoding *PPARG2, SREBF1, MOGAT2* and *DGAT1* were quantified by real-time PCR (7300 Real-Time PCR System; Applied Biosystems, Foster City, CA, USA). Primers and probes (Table S1) were designed using the software Primer Express 2.0 (Applied Biosystems) and purchased from Genosys (Sigma, St. Louis, MO, USA). Primers or TaqMan^®^ probes encompassing fragments of the areas from the extremes of two exons were designed to ensure the detection of the corresponding transcript avoiding genomic DNA amplification. The cDNA was amplified at the following conditions: 95 °C for 10 min, followed by 45 cycles of 15 s at 95 °C and 1 min at 59 °C, using the TaqMan^®^ Universal PCR Master Mix (Applied Biosystems). The primer and probe concentrations were 300 and 200 nmol/L, respectively. All results were normalized for *18S* rRNA expression (Applied Biosystems), and relative quantification was calculated using the 2^−∆∆Ct^ formula [47].

### 5.5. Immunohistochemistry of ANGPTL8

Sections of formalin-fixed paraffin-embedded human or rat liver (4 µm) were used to detect ANGPTL8 by the indirect immunoperoxidase assay [46]. Slides were deparaffinized in xylene and rehydrated in graded ethanol solutions. Endogenous peroxidase activity was blocked by incubation with 3% H_2_O_2_ (Sigma) in absolute methanol for 10 min at room temperature (RT), followed by washing in absolute ethanol. For epitope retrieval, sections were heated in a microwave oven for 20 min at 800 W and 400 W in a 10 mmol/L sodium citrate buffer (pH 6.00). Nonspecific binding was blocked with 1% murine serum (Sigma) diluted in Tris-buffer saline (TBS) (50 mmol/L Tris, 0.5 mol/L NaCl; pH 7.36) for 1 h at RT. Slides were incubated overnight at 4 °C with rabbit polyclonal anti-ANGPTL8 (SAB3501080, Sigma) diluted 1:100 in TBS. After washing the slides in TBS three times (5 min each), a pure DAKO Real^TM^ EnVision^TM^ anti-rabbit/mouse HRP polymer (K5007; Dako, Golstrup, Denmark) was added for 1 h at RT. The peroxidase reaction was visualized using a 0.5 mg/mL diaminobenzidine (DAB)/0.03% H_2_O_2_ solution diluted in 50 mmol/L Tris-HCl, pH 7.36 and Harris hematoxylin solution (Sigma) as counterstaining.

### 5.6. Cell Culture and Treatment

Human HepG2 hepatocytes (European Collection of Cell Cultures, Sigma) were seeded at 3 × 10^5^ cell/cm^2^ and grown in DMEM containing 10% FBS, 25 mmol/L D-glucose and 1% antibiotic-antimycotic (Invitrogen). Cells were serum-starved for 24 h and treated with palmitic acid (200 μmol/L) (P0500, Sigma) diluted in DMEM 5% BSA in the presence or absence of ANGPTL8 (10 ng/mL) (10159-AN, R&D systems) for 24 h. Palmitate concentration was chosen on the basis of prior experiments performed in our laboratory [45]. Intracellular TG content was measured by enzymatic methods, as earlier described [45].

### 5.7. Proliferation Assay

The proliferation of HepG2 hepatocytes was determined by measuring the amount of thymidine analog bromodeoxyuridin (BrdU) incorporated into nuclear DNA with the BrdU cell proliferation assay (QIA58, Calbiochem, Darmstadt, Germany) following the manufacturer’s instructions. The proliferative response was expressed as percentage of proliferation of treated cells compared to basal proliferation of unstimulated cells.

### 5.8. Statistical Analysis

Data are expressed as mean ± SEM. Differences between mean values were determined using a Student’s *t*-test, χ^2^ test, and two-way or one-way ANOVA followed by Scheffé’s, Tukey’s or Dunnet’s tests, where appropriate. Pearson’s correlation coefficients (r) and stepwise multiple linear regression analysis were used to analyze the association between variables using age, sex and BMI as confounding factors. A *p* value < 0.05 was considered statistically significant. Statistical analyses were analyzed with SPSS 15.0.

## Figures and Tables

**Table 1:** Clinical characteristics of the participants enrolled in the study.

	Lean	Obese NG	Obese IGT	Obese T2D	*p*
*n*	30	48	47	45	-
Sex (male/female)	16/14	17/31	17/30	20/25	0.199
Age (years)	43 ± 3	40 ± 2	44 ± 2	48 ± 2 ^b^	**0.031**
BMI (kg/m^2^)	22.6 ± 0.5	44.5 ± 1.3 ^a^	43.2 ± 0.9 ^a^	45.4 ± 1.4 ^a^	**<0.0001**
Body fat (%)	21.2 ± 1.4	50.7 ± 0.9 ^a^	51.0 ± 1.3 ^a^	50.1 ± 1.2 ^a^	**<0.0001**
Waist circumference (cm)	77 ± 3	125 ± 2 ^a^	125 ± 2 ^a^	132 ± 3 ^a^	**<0.0001**
Glucose (mg/dL)	87 ± 2	92 ± 1	105 ± 1 ^a,b^	142 ± 9 ^a,b^	**<0.0001**
Glucose 2-h OGTT (mg/dL)	-	121 ± 4	154 ± 5 ^b^	250 ± 16 ^b^	**<0.0001**
Insulin (µU/mL)	5.7 ± 0.6	20.9 ± 2.7 ^a^	19.6 ± 1.7 ^a^	26.3 ± 3.1 ^a^	**<0.0001**
Insulin 2-h OGTT (µU/mL)	-	97.0 ± 9.5	141.1 ± 12.4 ^b^	133.3 ± 19.2 ^b^	**0.034**
HOMA	1.2 ± 0.1	4.3 ± 0.4	5.2 ± 0.5 ^a^	9.2 ± 1.7 ^a,b^	**<0.0001**
QUICKI	0.38 ± 0.01	0.32 ± 0.01 ^a^	0.31 ± 0.01 ^a^	0.30 ± 0.01 ^a,b^	**<0.0001**
FFA (mmol/L)	15.7 ± 0.1	16.7 ± 1.1	22.5 ± 2.2	28.9 ± 4.0 ^a,b^	**0.001**
Glycerol (mg/dL)	18.2 ± 3.5	19.8 ± 2.1	22.1 ± 2.5	21.9 ± 2.3	0.591
Adipo-IR index	20.2 ± 3.4	71.3 ± 7.7	98.9 ± 13.7	188.4 ± 36.3 ^a,b^	**<0.0001**
Triacylglycerol (mg/dL)	70 ± 5	111 ± 7 ^a^	132 ± 11 ^a^	147 ± 11 ^a,b^	**<0.0001**
Total cholesterol (mg/dL)	186 ± 7	191 ± 4	203 ± 6	198 ± 6	0.234
LDL cholesterol (mg/dL)	110 ± 6	118 ± 3	127 ± 5	116 ± 5	0.145
HDL cholesterol (mg/dL)	61 ± 3	50 ± 4	49 ± 2 ^a^	44 ± 2 ^a^	**0.001**
CRP (mg/L)	1.8 ± 0.3	8.3 ± 1.1	9.5 ± 1.5 ^a^	8.8 ± 1.8 ^a^	**0.035**
Uric acid (mg/dL)	4.3 ± 0.2	5.7 ± 0.2 ^a^	5.9 ± 0.2 ^a^	6.0 ± 0.2 ^a^	**0.001**
Leptin (ng/mL)	6.1 ± 0.7	45.4 ± 3.2 ^a^	48.9 ± 3.6 ^a^	46.0 ± 5.8 ^a^	**<0.0001**
AST (IU/L)	13 ± 1	16 ± 1	17 ± 1	17 ± 1	0.244
ALT (IU/L)	10 ± 2	21 ± 2	28 ± 2 ^a^	27 ± 2 ^a^	**<0.0001**
Alkaline phosphatase (IU/L)	86 ± 5	68 ± 5	77 ± 6	73 ± 5	0.334
γ-GT (IU/L)	11 ± 1	21 ± 2	29 ± 4 ^a^	30 ± 4 ^a^	**0.015**
Daily alcohol intake (g)	0.0 ± 0.0	1.1 ± 0.9	3.6 ± 2.7	4.6 ± 2.7	0.576
Antihypertensive therapy, n (%)	0 (0%)	11 (23%)	17 (32%)	19 (49%)	**0.003**
Antidiabetic therapy, n (%)	0 (0%)	2 (4%)	1 (2%)	20 (51%)	**<0.0001**
Lipid-lowering therapy, n (%)	0 (0%)	6 (13%)	7 (15%)	11 (28%)	0.061

NG, normoglycemia; IGT, impaired glucose tolerance; T2D, type 2 diabetes; BMI, body mass index; OGTT, oral glucose tolerance test; HOMA, homeostasis model assessment; QUICKI, quantitative insulin sensitivity check index; FFA, free fatty acids; Adipo-IR, adipocyte insulin resistance index; CRP, high-sensitivity C-reactive protein; AST, aspartate aminotransferase; ALT, alanine aminotransferase; γ-GT, γ-glutamyltransferase. Differences between groups were analyzed by one-way ANOVA followed by a Scheffe’s test or χ^2^ test, where appropriate. Bold values denote statistically significant *p* values. ^a^ *p* < 0.05 vs. normal-weight individuals; ^b^ *p* < 0.05 vs. obese NG patients.

**Table 2:** Effect of weight loss induced by bariatric surgery on clinical characteristics of patients with morbid obesity.

	Sleeve Gastrectomy (*n* = 34)			Roux-en-Y Gastric Bypass (*n* = 41)		
	Pre-Surgery	Post-Surgery	*p*	Pre-Surgery	Post-Surgery	*p*
Sex (male/female)	10/24	10/24	-	15/26	15/26	-
BMI (kg/m^2^)	41.0 ± 1.7	31.1 ± 1.6	**<0.0001**	44.7 ± 0.8	31.9 ± 0.6	**<0.0001**
Body fat (%)	50.9 ± 1.2	39.5 ± 1.8	**<0.0001**	50.7 ± 0.8	38.1 ± 1.0	**<0.0001**
Waist circumference (cm)	120 ± 3	98 ± 3	**<0.0001**	127 ± 2	100 ± 2	**<0.0001**
Glucose (mg/dL)	106 ± 9	92 ± 4	**0.022**	115 ± 5	95 ± 4	**<0.0001**
Insulin (µU/mL)	30.9 ± 6.6	10.0 ± 1.6	**0.005**	23.0 ± 3.3	8.6 ± 1.1	**<0.0001**
HOMA	8.3 ± 2.1	2.1 ± 0.4	**0.011**	6.2 ± 1.3	2.3 ± 0.6	**<0.0001**
QUICKI	0.30 ± 0.01	0.36 ± 0.01	**<0.0001**	0.31 ± 0.01	0.36 ± 0.01	**<0.0001**
FFA (mmol/L)	18.8 ± 1.2	14.3 ± 1.5	0.052	21.5 ± 2.2	13.2 ± 1.0	**0.001**
Glycerol (mg/dL)	19.4 ± 2.2	14.0 ± 0.8	**0.043**	22.4 ± 2.1	12.9 ± 1.3	**0.001**
Adipo-IR index	138.7 ± 40.0	32.6 ± 6.3	**0.020**	91.9 ± 12.7	28.7 ± 5.6	**<0.0001**
Triacylglycerol (mg/dL)	110 ± 15	98 ± 10	0.380	136 ± 9	90 ± 4	**<0.0001**
Total cholesterol (mg/dL)	185 ± 8	186 ± 9	0.919	199 ± 5	158 ± 4	**<0.0001**
LDL cholesterol (mg/dL)	115 ± 7	119 ± 7	0.576	122 ± 5	92 ± 3	**<0.0001**
HDL cholesterol (mg/dL)	47 ± 3	52 ± 3	0.111	49 ± 3	48 ± 1	0.666
CRP (mg/L)	4.6 ± 1.5	3.0 ± 1.2	0.252	7.9 ± 1.1	2.4 ± 0.3	**<0.0001**
Uric acid (mg/dL)	5.0 ± 0.3	4.7 ± 0.3	0.203	6.1 ± 0.2	4.9 ± 0.1	**<0.0001**
Leptin (ng/mL)	77.0 ± 12.7	25.3 ± 5.9	**0.004**	51.0 ± 4.6	16.2 ± 2.5	**<0.0001**
AST (IU/L)	17 ± 1	17 ± 1	0.983	18 ± 1	18 ± 1	0.849
ALT (IU/L)	22 ± 3	19 ± 2	0.318	27 ± 2	26 ± 2	0.606
Alkaline phosphatase (IU/L)	64 ± 6	65 ± 5	0.916	82 ± 5	84 ± 5	0.529
γ-GT (IU/L)	29 ± 6	22 ± 4	**0.027**	24 ± 3	14 ± 2	**<0.0001**
Antihypertensive therapy, *n* (%)	15 (44%)	12 (34%)	**0.001**	17 (41%)	9 (22%)	**0.011**
Antidiabetic therapy, *n* (%)	8 (24%)	5 (15%)	0.085	12 (29%)	4 (10%)	**0.001**
Lipid-lowering therapy, *n* (%)	8 (24%)	3 (9%)	0.113	11 (27%)	3 (7%)	0.081

BMI, body mass index; HOMA, homeostasis model assessment; QUICKI, quantitative insulin sensitivity check index; FFA, free fatty acids; Adipo-IR, adipocyte insulin resistance index; CRP, high-sensitivity C-reactive protein; AST, aspartate aminotransferase; ALT, alanine aminotransferase; γ-GT, γ-glutamyltransferase. Differences between groups were analyzed by paired two-tailed Wilcoxon *t*-test or χ^2^ test, where appropriate. Bold values denote statistically significant *p* values.

## Data Availability

The data presented in this study are available on request from the corresponding author. The data are not publicly available due to privacy restrictions.

## Supplementary Materials

The following are available online at https://www.mdpi.com/article/10.3390/ijms222312945/s1.
