# Supplementary material for: Role of ANGPTL8 in NAFLD Improvement after Bariatric Surgery in Experimental and Human Obesity
Source: Int J Mol Sci. 2021 Nov 30;22(23):12945. doi: 10.3390/ijms222312945 (PMC8657645; doi:10.3390/ijms222312945)
Supplement: Supplementary file 1 [file ijms-22-12945-s001.zip › ijms-1473865-supplementary.pdf]

Table S1. Sequences of primers and TaqMan® probes.

Gene (GenBank accesion no.)	Oligonucleotide sequence (5′-3′)	Nucleotides
**Rat**		
Angptl8 (NM_001271710.1)		
Forward	TTGTCGGAGATTCAGGCAGAA	304-324
Reverse	GAACAGTGTACGGAGACTACAAGTGC	402-427
Probe	FAM-TACACCTTCGAGCAGAA-TAMRA	334-351
**Human**		
ANGPTL8 (NM_018687.7)		
Forward	TCTTAAAGGCTCACGCTGACAA	471-492
Reverse	ATGGTGGCACAGCAGCATC	548-566
Probe	FAM-AGCCACATCCTATGGGCCCTCACAG-TAMRA	497-521
HNF4A (NM_000457.5)		
Forward	GTGCGTGGTGGACAAAGACA	437-456
Reverse	GAAGAAGGAAGCCGTCCAGAA	509-529
Probe	FAM-CTCAAGAAATGCTTCCGGGCTGGCA-TAMRA	483-507
DGAT1 (NM_012079.6)		
Forward	GCTACCCGGACAATCTGACCTA	941-962
Reverse	TTGAGATGCTGTTCTTCACCCA	1064-1085
Probe	FAM-TACTTCCTCTTCGCCCCCACCTTGTG-TAMRA	971-1001
MOGAT2 (NM_025098.4)		
Forward	TTCGATCTTCCCCGGTATCC	438-457
Reverse	TGTCTGCAGGGTTGGTCACA	518-537
Probe	FAM-ATGATGCTGACCTTGTGGTTCCGGG-TAMRA	469-493
PPARG2 (NM_015869.4)		
Forward	AGCCTCATGAAGAGCCTTCCA	465-485
Reverse	TTGTGAAGGATGCAAGGGTTTC	556-577
Probe	FAM-TCCCTCATGGCAATTGAATGTCGTGTC-TAMRA	488-514
SREBF1 (NM_004176.5)		
Forward	ACATCGAAGACATGCTTCAGCTT	252-274
Reverse	TTTGACCCACCCTATGCTGG	308-327
Probe	FAM-CAACAACCAAGACAGTGACTTCCCTGGC-TAMRA	277-304

ANGPTL8, angiopoietin-like protein 8; *DGAT1,* diacylglycerol *O*-acyltransferase 1; *HNF4A*, hepatocyte nuclear factor 4 α; *MOGAT2*, monoacylglycerol *O*-acyltransferase 2; *PPARG2*, peroxisome proliferator-activated receptor γ 2; *SREBF1*, sterol regulatory element-binding transcription factor 1.

Table S2. Multiple linear regression analyses with plasma ANGPTL8 as dependent variable for all the participants in the cross-sectional study.

	Plasma ANGPTL8	
Model I	β	*P*
Age	-0.001	0.994
Sex	-4.889	0.115
BMI	-0.009	0.947
HOMA	-1.104	**0.003**
Adipo-IR	0.025	0.088
Insulin	0.217	0.082
Adjusted R^2	0.101	**0.049**
Model II	β	*P*
Age	-0.070	0.671
Sex	-8.695	**0.025**
BMI	0.346	0.180
γ-GT	-0.231	**0.045**
Hepatic steatosis	-2.294	0.350
Adjusted R^2	0.110	**0.046**

BMI, body mass index; HOMA, homeostasis model assessment. The coefficient of dichotomous variable Sex from the regression represents the effect of male relative to female. β means standardized regression beta coefficients. Adjusted R^2 expresses the percentage of the variance explained by the independent variables in the different models (i.e. 0.101 is 10.1%). Statistical significant values are in bold.

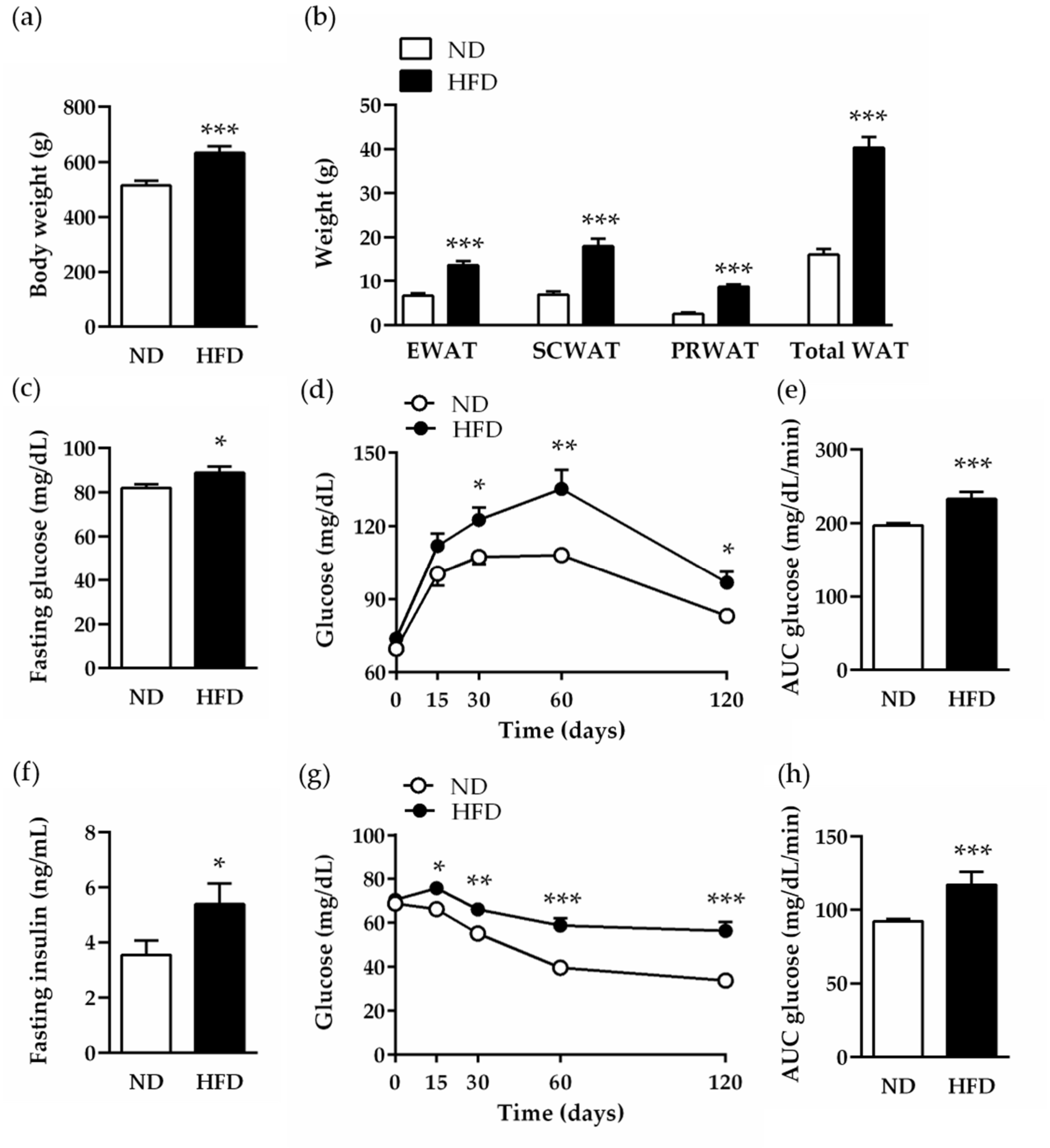

Figure S1. Increased body weight, whole-body adiposity, impaired glucose tolerance and insulin resistance in rats with diet-induced obesity. Bar graphs show the (a) body weight, (b) epididymal (EWAT), subcutaneous (SCWAT), perirenal (PRWAT) and total white fat content as well as fasting serum (c) glucose and (f) insulin of lean and diet-induced obese rats. Blood glucose levels and area under the curve (AUC) during OGTT (d and e) and IPITT (g and h) in rats fed a normal diet (ND) or a high-fat diet (HFD). Statistical differences were analyzed by using a Student's *t* test. *P<0.05, **P<0.01, ***P<0.001 *vs* control rats fed a ND.

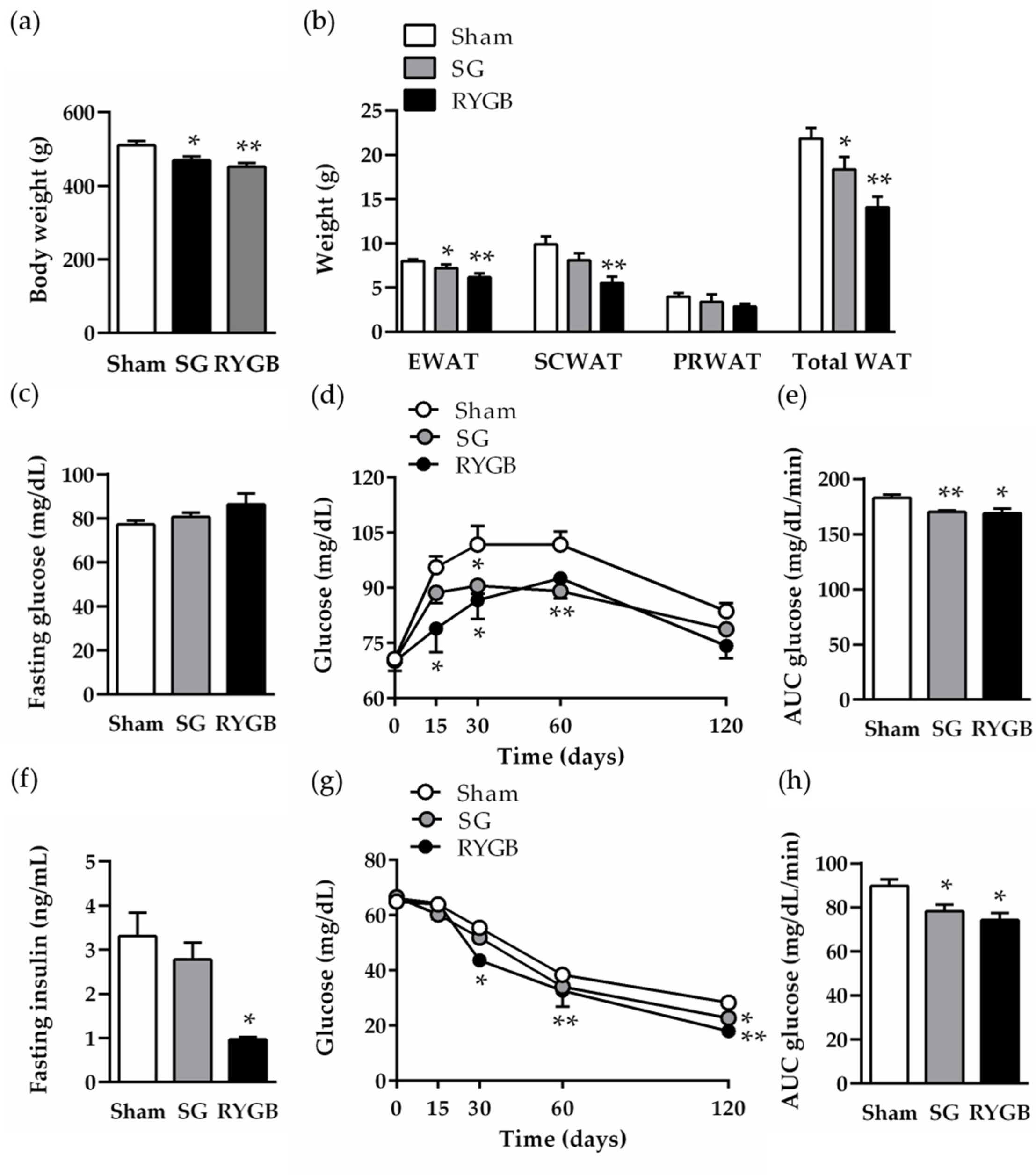

Figure S2. Bariatric surgery improved body weight, whole-body adiposity, glucose tolerance and insulin sensitivity of rats with diet-induced obesity. Bar graphs show the (a) body weight, (b) epididymal (EWAT), subcutaneous (SCWAT), perirenal (PRWAT) and total white fat content as well as fasting serum (c) glucose and (f) insulin of rats with diet-induced obesity one month after sham surgery, sleeve gastrectomy (SG) or Roux-en-Y gastric bypass (RYGB). Blood glucose levels and area under the curve (AUC) during OGTT (d and e) and IPITT (g and h) in obese rats submitted to sham surgery, SG or RYGB. Statistical differences were analyzed by one-way ANOVA followed by a Tukey's test. *$P<0.05$, **$P<0.01$ *vs* obese rats submitted to sham surgery.
